# Effects of wood ash and N fertilization on soil chemical properties and growth of *Zelkova serrata* across soil types

**DOI:** 10.1038/s41598-021-93805-5

**Published:** 2021-07-14

**Authors:** Ji Young An, Byung Bae Park

**Affiliations:** 1 Institute of Agricultural Science, College of Agriculture and Life Sciences, Chungnam National University, Daejeon, 34134 Republic of Korea; 2 Department of Forest and Environmental Sciences, Chungnam National University, Daejeon, 34134 Republic of Korea; 3 Department of Environment and Forest Resources, College of Agriculture and Life Sciences, Chungnam National University, Daejeon, 34134 Republic of Korea

**Keywords:** Plant sciences, Environmental sciences, Forest ecology, Forestry, Carbon cycle

## Abstract

Wood ash generated as a by-product of biomass combustion can be a sustainable and reasonable approach to counteract acidification and correct nutrient deficiency in forest soils. We investigated the influence of wood ash (WA) and combined WA + N (nitrogen) on soil chemical properties, growth and foliar nutrients of *Zelkova serrata* and their potential as a soil amender across different soil types. We applied four levels of WA (0, 5, 10, and 20 Mg ha^−1^) and two levels of N fertilizer (0 and 150 kg ha^−1^) across three different soil types: landfill saline (LS) soil, forest infertile (FI) soil, and forest acidic (FA) soil. The WA generally improved soil pH, organic matter, available P, exchangeable cations (K^+^, Na^+^, Ca^2+^, and Mg^2+^), and EC of the three soils, but its ameliorating and neutralizing effects were predominant in FA soil. N fertilizer was more effective in improving plant growth, especially for biomass production in LS and FI soils. WA application significantly increased biomass production when it was applied over 5 Mg ha^−1^ in FA soil, but higher dose rate of WA (i.e. 20 Mg ha^−1^) seems to pose negative effects. Foliar P, K, and Ca concentrations also tended to increase with the increasing amount of WA. Therefore, lower dosage of WA without N can be applied as a soil amender to counteract forest soil acidity and improve plant growth and foliar nutrient concentration, whereas N fertilizer without WA can be added to correct nutrient soil deficiencies in landfill and infertile soils. This study should enhance our understanding of WA as a sustainable and reasonable approach to counteract acidification and correct nutrient deficiency in forest soils.

## Introduction

Many of the proposed goals related to clean energy, food, climate, and terrestrial biodiversity and ecosystems in the Sustainable Development Goals (SDGs) of the united nations are reliant on biomass^1,2^. However, the practical and explicit approaches to sustainable biomass production and consumption are still lacking and, therefore, should be one of the foci of research^1^. Biomass combustion is one of the promising sustainable practices for CO_2_-neutral heat and power generation when, for example, the by-products such as wood ash, are reutilized and managed appropriately^3,4^. Increased exploitation of wood biomass to meet the heat and power market demands, however, poses environmental risks and threatens forest and land productivity. The increased interest in bioenergy has also led to more intense whole-tree harvests in forest plantations^5^, making forest and land productivity unsustainable through depleting the nutrient reserves in soils. Whole-tree harvesting has already been reported to deplete soil calcium (Ca) levels which negatively affect tree growth^6,7^ and influence soil pH with consistent effects on nutrient availability, particularly nitrogen^8^. Thus, a continuing discussion on effective strategies for balancing the pros and cons of biomass combustion is needed as investment in bioenergy increases.

Previous studies have already shown that wood ash, often considered as waste material, can be used as a liming agent and soil amendment for nutrient deficiency or imbalances in forests^9^. Several studies have illustrated increases in soil pH, nutrient content^10–12^ and microbial activity, mineralization, and nitrification^13,14^ from ash applications. Both field and greenhouse experiments have also shown that the improvements in physical, chemical, and biological soil properties^12,15,16^ from ash applications have resulted in a significant increase in plant growth^17,18^. However, while it already contains most of the desirable macronutrients (e.g. Ca, P, K, and Mg), nitrogen (N) is very limited in wood ash^19–21^ because N is lost through the burning process. Therefore, wood ash alone application may not be effective to compensate for deficiency of N^22–24^ , which is generally considered to be limited in soils across the globe^25^ and the main nutrient limiting forest growth on mineral soils^26^. Soil N availability has also been projected to be more limited due to global climate change^27^. Thus, there is a need to advance our understanding on how wood ash can be used as a total nutrient management approach because the absence of N in ash makes soil amendment only a minor part of the total soil fertility management. There have been several studies in which the effects of combined wood ash + N on soil chemical properties and plant growth have been investigated. For example, a combination of wood ash and urea-N fertilizer resulted in a significantly higher, not only the basal area of trees, but also the soil concentrations of N, Ca, K, and Mg and rate of net N mineralization compared with the control and ash/N alone treatments^22^. However, the effects of both wood ash alone and wood ash + N application seem to be very complex. Solla-Gullón et al.^28^ reported that increases in height and diameter growth of *Pinus radiata* D. Don seedlings were attributed to increased concentrations of Ca and Mg in ash-amended soil, while Demeyer et al.^19^ attributed the growth response to increased availability of K, P, and B in the soil after ash application. Similarly, wood ash + N application was found to be less responsive than N alone application, and the former treatment resulted in no extra growth response^13^. Contrarily, a positive effect of wood ash + N on growth was detected when applied on a less-productive mineral soil site^29^, suggesting that soil types or the initial amount of soil nutrients, particularly N, may influence the response to wood ash + N application. The variation in responses of different soil types to wood ash soil amendment may already be obvious, but as to the magnitude of the effect and amount of wood ash + N to reduce acidity or correct soil nutrient deficiencies remain unclear to date.

Consequently, we aimed at determining the effects of wood ash and N fertilization on soil chemical properties, growth and foliar nutrients of *Zelkova serrata* (Thunb.) Makino and its potential to ameliorate saline, infertile, and acidic soils. The primary question in this study was whether wood ash could ameliorate the said soil types and the addition of N fertilizer would give better tree growth and foliar nutrients regardless of soil types. Thus, we hypothesized that (1) the effect of wood ash as a soil ameliorant varies by soil types and, (2) the addition of N would compensate the N deficiency in wood ash resulting in higher tree biomass growth and foliar nutrients in all soil types. This study should provide us better understanding of the use of wood ash soil amendment for improving plant, forest, and soil productivity in a sustainable way.

## Results

### Change of soil chemical properties

The application of wood ash and N fertilizer resulted in significant changes to soil chemical properties in all soils, and the magnitude of response to treatments was different across soil types (Table 1). Over 5 Mg ha^−1^ wood ash (5 WA) dose ameliorated soil acidity and soil pH increased as wood ash amount increased in FA soil. WA also increased the pH of LS and FI soil, but this increase was similar across different amounts (5 to 20 Mg ha^−1^) of added WA compared with the control (0WA + 0N). Available P increased as the amount of WA increased in all soil types while CEC was not influenced by wood ash application except FA soil. Exchangeable K^+^ and Ca^2+^ positively responded to wood ash application resulting in the highest values in 20 WA treatment in all soil types; however, exchangeable Mg^2+^ showed decreasing pattern in LS soil and increasing pattern in FI and FA soil by adding wood ash. Wood ash application increased EC in all soil types but the amount of applied wood ash was less influential on it. NaCl was affected by neither wood ash nor N fertilizer.

**Table 1:** Soil chemical properties applied with four levels of wood ash (WA; 0, 50, 10, and 20 Mg ha^−1^) and two levels of N fertilizer (N fert; 0 and 150 kg ha^−1^) across three different soil types (LS, landfill saline soil; FI, forest infertile soil; FA, forest acidic soil) at the end of this study (20 weeks after treatment).

Soil	N fert (kg ha^−1^)	Wood ash (Mg ha^−1^)	pH	Organic matter (%)	Total N (g kg^−1^)	Available P (mg kg^−1^)	CEC (cmolc kg^−1^)	
LS	0	0	8.7 (0.0)^b^	0.83 (0.03)^b^	0.21 (0.01)^a^	46.8 (1.0)^c^	15.8 (0.1)^a^	
		5	9.1 (0.1)^a^	0.97 (0.00)^b^	0.23 (0.01)^a^	57.7 (1.9)^bc^	13.6 (0.4)^a^	
		10	9.2 (0.1)^a^	1.37 (0.21)^ab^	0.21 (0.02)^a^	64.6 (1.1)^b^	15.3 (3.1)^a^	
		20	9.3 (0.1)^a^	1.97 (0.34)^a^	0.22 (0.00)^a^	112.0 NA^a^	12.4 (0.0)^a^	
	150	0	8.7 (0.1)^b^	0.81 (0.01)^b^	0.24 (0.01)^a^	46.6 (0.8)^c^	14.6 (0.9)^a^	
		5	9.0 (0.0)^ab^	0.97 (0.06)^b^	0.24 (0.03)^a^	55.8 (1.1)^bc^	12.7 (0.9)^a^	
		10	9.2 (0.1)^a^	1.16 (0.14)^ab^	0.23 (0.02)^a^	66.4 (3.6)^b^	13.6 (0.2)^a^	
		20	9.2 (0.1)^a^	1.45 (0.07)^ab^	0.22 (0.01)^a^	108.5 (2.5)^a^	14.8 (2.5)^a^	
		p values						
		WA	**0.0001**	**0.002**	0.82	** < 0.0001**	0.55	
		N	0.25	0.12	0.16	0.54	0.76	
		WA * N	0.64	0.35	0.76	0.64	0.54	
FI	0	0	7.6 (0.2)^b^	0.33 (0.01)^d^	0.07 (0.01)^a^	17.4 (0.4)^c^	9.3 (0.5)^a^	
		5	8.5 (0.0)^a^	0.44 (0.00)^cd^	0.08 (0.02)^a^	34.6 (0.8)^bc^	8.7 (0.4)^a^	
		10	8.7 (0.1)^a^	0.76 (0.04)^b^	0.09 (0.00)^a^	53.1 (1.0)^b^	8.2 (0.6)^a^	
		20	8.8 (0.0)^a^	1.04 (0.09)^a^	0.10 (0.02)^a^	88.8 (5.6)^a^	7.4 (0.2)^a^	
	150	0	7.4 (0.1)^b^	0.34 (0.03)^d^	0.09 (0.02)^a^	22.9 (6.9)^c^	9.1 (0.2)^a^	
		5	8.5 (0.1)^a^	0.48 (0.00)^cd^	0.09 (0.02)^a^	35.0 (1.4)^bc^	8.9 (1.4)^a^	
		10	8.7 (0.0)^a^	0.68 (0.02)^bc^	0.09 (0.01)^a^	54.7 (4.0)^b^	8.7 (0.7)^a^	
		20	9.0 (0.0)^a^	1.10 (0.07)^a^	0.09 (0.01)^a^	91.5 (3.9)^a^	8.7 (1.1)^a^	
		p values						
		WA	** < 0.0001**	** < 0.0001**	0.62	** < 0.0001**	0.49	
		N	0.94	0.84	0.67	0.37	0.42	
		WA * N	0.29	0.45	0.73	0.91	0.77	
FA	0	0	4.9 (0.1)^d^	3.29 (0.01)^a^	0.83 (0.05)^a^	4.1 (0.9)^c^	8.7 (0.3)^b^	
		5	6.4 (0.1)^c^	3.19 (0.07)^a^	0.72 (0.02)^ab^	7.3 (0.6)^c^	7.9 (0.0)^b^	
		10	7.6 (0.0)^b^	3.00 (0.06)^a^	0.50 (0.04)^b^	16.6 (2.2)^b^	6.8 (0.7)^b^	
		20	8.2 (0.1)^a^	3.08 (0.14)^a^	0.50 (0.02)^b^	27.4 NA^a^	14.7 (0.7)	
	150	0	4.6 (0.1)^d^	3.24 (0.04)^a^	0.91 (0.10)^a^	3.9 (0.1)^c^	9.4 (0.1)^b^	
		5	6.2 (0.1)^c^	3.22 (0.08)^a^	0.74 (0.07)^ab^	7.9 (0.6)^c^	8.3 (0.6)^b^	
		10	7.8 (0.0)^b^	2.95 (0.04)^a^	0.64 (0.08)^ab^	17.0 (1.3)^b^	7.6 (0.1)^b^	
		20	8.2 (0.0)^a^	3.20 (0.00)^a^	0.61 (0.05)^ab^	28.3 (1.6)^a^	15.0 (1.8)^a^	
		p values						
		WA	** < 0.0001**	**0.01**	**0.002**	** < 0.0001**	** < 0.0001**	
		N	0.09	0.78	0.06	0.66	0.37	
		WA * N	**0.01**	0.54	0.78	0.98	0.98	

Soil	N fert (kg ha^−1^)	Wood ash (Mg ha^−1^)	Exchangeable cations				EC (dS m^−1^)	NaCl (%)
			K^+^ (cmolc kg^−1^)	Na^+^ (cmolc kg^−1^)	Ca^2+^ (cmolc kg^−1^)	Mg^2+^ (cmolc kg^−1^)		
LS	0	0	0.95 (0.05)^d^	3.18 (0.08)^a^	2.91 (0.19)^cd^	5.09 (0.23)^a^	0.69 (0.03)^b^	0.022 (0.002)^a^
		5	1.47 (0.04)^cd^	2.82 (0.44)^a^	6.86 (0.78)^bc^	5.04 (0.02)^a^	1.01 (0.06)^ab^	0.021 (0.002)^a^
		10	2.21 (0.29)^bc^	2.28 (0.52)^a^	8.52 (0.20)^ab^	4.74 (0.26)^ab^	1.08 (0.15)^ab^	0.024 (0.003)^a^
		20	3.79 (0.32)^a^	2.20 (0.08)^a^	11.12 (0.14)^a^	3.50 (0.00)^c^	1.12 (0.01)^a^	0.023 (0.001)^a^
	150	0	0.98 (0.07)^d^	2.90 (0.03)^a^	2.77 (0.19)^d^	5.23 (0.10)^a^	0.75 (0.09)^ab^	0.025 (0.003)^a^
		5	1.43 (0.12)^cd^	1.89 (0.25)^a^	7.49 (0.53)^ab^	4.80 (0.04)^a^	0.85 (0.02)^ab^	0.019 (0.001)^a^
		10	1.77 (0.06)^cd^	2.26 (0.12)^a^	9.90 (0.87)^ab^	4.62 (0.29)^ab^	1.04 (0.00)^ab^	0.023 (0.001)^a^
		20	3.11 (0.15)^ab^	1.92 (0.29)^a^	11.46 (1.59)^a^	3.87 (0.04)^bc^	1.00 (0.09)^ab^	0.018 (0.001)^a^
		p values						
		WA	** < 0.0001**	**0.04**	** < 0.0001**	** < 0.0001**	**0.004**	0.15
		N	**0.046**	0.09	0.32	0.75	0.25	0.32
		WA * N	0.20	0.46	0.77	0.32	0.47	0.18
FI	0	0	0.14 (0.01)^d^	0.12 (0.01)^a^	4.39 (0.46)^d^	0.64 (0.03)^d^	0.22 (0.00)^c^	0.014 (0.001)^a^
		5	0.34 (0.01)^cd^	0.11 (0.00)^a^	7.31 (0.27)^bcd^	0.62 (0.01)^d^	0.38 (0.01)^abc^	0.017 (0.000)^a^
		10	0.75 (0.04)^b^	0.12 (0.01)^a^	9.91 (0.58)^ab^	0.80 (0.01)^cd^	0.40 (0.01)^ab^	0.017 (0.000)^a^
		20	1.62 (0.14)^a^	0.13 (0.01)^a^	11.75 (0.14)^a^	1.03 (0.01)^ab^	0.53 (0.08)^a^	0.018 (0.002)^a^
	150	0	0.16 (0.02)^d^	0.12 (0.01)^a^	4.82 (0.41)^cd^	0.79 (0.02)^cd^	0.23 (0.05)^bc^	0.015 (0.000)^a^
		5	0.43 (0.02)^bcd^	0.12 (0.00)^a^	7.88 (0.46)^bc^	0.63 (0.00)^d^	0.38 (0.01)^abc^	0.015 (0.002)^a^
		10	0.63 (0.04)^bc^	0.15 (0.01)^a^	11.47 (1.43)^a^	0.90 (0.09)^bc^	0.43 (0.01)^a^	0.018 (0.000)^a^
		20	1.53 (0.10)^a^	0.16 (0.02)^a^	12.46 (0.25)^a^	1.10 (0.03)^a^	0.54 (0.01)^a^	0.017 (0.001)^a^
		p values						
		WA	** < 0.0001**	**0.049**	** < 0.0001**	** < 0.0001**	** < 0.0001**	0.06
		N	0.59	**0.03**	0.10	**0.007**	0.62	0.72
		WA * N	0.39	0.33	0.80	0.28	0.96	0.37
FA	0	0	0.19 (0.03)^d^	0.23 (0.01)^ab^	1.10 (0.25)^d^	0.23 (0.02)^bc^	0.27 (0.01)^ab^	0.016 (0.001)^a^
		5	0.98 (0.09)^cd^	0.24 (0.00)^a^	5.39 (0.02)^c^	0.41 (0.01)^ab^	0.46 (0.13)^a^	0.016 (0.000)^a^
		10	1.57 (0.01)^bc^	0.22 (0.01)^ab^	8.49 (0.67)^b^	0.26 (0.02)^abc^	0.63 (0.00)^ab^	0.018 (0.001)^a^
		20	3.01 (0.25)^a^	0.22 (0.01)^ab^	13.77 (0.73)^a^	0.48 (0.11)^a^	0.86 (0.02)^a^	0.018 (0.002)^a^
	150	0	0.15 (0.01)^d^	0.19 (0.01)^b^	0.77 (0.02)^d^	0.18 (0.01)^c^	0.60 (0.03)^c^	0.014 (0.000)^a^
		5	0.97 (0.16)^cd^	0.24 (0.00)^a^	4.79 (0.30)^c^	0.42 (0.01)^ab^	0.78 (0.04)^bc^	0.017 (0.000)^a^
		10	1.96 (0.24)^b^	0.25 (0.01)^a^	10.09 (0.64)^b^	0.29 (0.00)^abc^	0.71 (0.00)^ab^	0.019 (0.001)^a^
		20	2.90 (0.27)^a^	0.24 (0.01)^a^	14.12 (0.69)^a^	0.38 (0.02)^abc^	0.82 (0.01)^a^	0.016 (0.001)^a^
		p values						
		WA	** < 0.0001**	**0.007**	** < 0.0001**	**0.001**	**0.0002**	**0.03**
		N	0.63	0.65	0.49	0.37	**0.0009**	0.42
		WA * N	0.47	**0.007**	0.20	0.39	**0.01**	0.27

Standard errors are shown in parentheses (n = 2). P values are from the result of two-way ANOVA to detect the effect of wood ash and N fertilizer on each soil property within each soil type. Significant p values are in bold. Different lower case letters within each soil indicate significant differences (p < 0.05, Tukey–Kramer comparison lines for least squares means) in each soil property across four wood ash levels and N fertilizer treatment.

### Plant growth and biomass allocation

Overall, N fertilizer increased plant height growth while the wood ash application was effective for RCD growth in all soil types (Table 2). Plant height and RCD growth increased the most at 10 WA application in LS soil. Meanwhile, N fertilizer increased plant height by 44% and RCD by 45% in FI soil, resulting in the highest RCD growth in 5 WA + N. In FA soil, the application of 5 WA increased RCD growth of plant by 21% compared with plants that received no WA whereas N fertilizer enhanced height growth by 11% regardless of WA application.

**Table 2:** Growth of *Zelkova serrata* seedlings applied with four levels of wood ash (WA; 0, 5, 10, and 20 Mg ha^−1^) and two levels of N fertilizer (N fert; 0 and 150 kg ha^−1^) across three different soil types (LS, landfill saline soil; FI, forest infertile soil; FA, forest acidic soil) at the end of this study (20 weeks after treatment).

Soil	N fert (kg ha^−1^)	Wood ash (Mg ha^−1^)	Height (cm)	RCD (mm)	Leaf area (cm^2^ tree^−1^)	SLA (cm^2^ g^−1^)
LS	0	0	117 (8)^a^	9.6 (0.5)^a^	53 (6)^c^	65 (6)^a^
		5	98 (10)^a^	9.7 (0.5)^a^	63 (3)^bc^	67 (6)^a^
		10	112 (14)^a^	11.6 (1.6)^a^	59 (6)^bc^	68 (7)^a^
		20	108 (8)^a^	8.2 (0.5)^a^	45 (9)^c^	62 (10)^a^
	150	0	121 (8)^a^	10.2 (0.9)^a^	93 (3)^abc^	76 (1)^a^
		5	129 (6)^a^	10.8 (0.5)^a^	103 (20)^ab^	75 (7)^a^
		10	139 (3)^a^	12.1 (1.0)^a^	130 (14)^a^	90 (1)^a^
		20	117 (15)^a^	9.2 (0.7)^a^	63 (13)^bc^	70 (8)^a^
		p values				
		WA	0.67	**0.02**	**0.01**	0.35
		N	**0.01**	0.27	** < 0.0001**	**0.01**
		WA * N	0.30	0.97	0.14	0.75
FI	0	0	84 (7)^bcd^	8.0 (0.3)^d^	35 (4)^c^	54 (3)^a^
		5	117 (15)^abcd^	8.5 (0.6)^cd^	51 (8)^bc^	59 (4)^a^
		10	87 (2)^cd^	8.6 (0.3)^d^	39 (4)^c^	52 (4)^a^
		20	84 (4)^c^	8.2 (0.5)^d^	38 (5)^c^	56 (4)^a^
	150	0	130 (11)^ab^	11.9 (1.2)^ab^	136 (25)^a^	68 (4)^a^
		5	123 (11)^abc^	13.2 (0.6)^a^	112 (11)^a^	70 (4)^a^
		10	146 (11)^a^	12.3 (0.4)^ab^	120 (18)^ab^	68 (8)^a^
		20	135 (8)^a^	10.8 (0.8)^bc^	79 (17)^abc^	69 (9)^a^
		p values				
		WA	0.41	**0.02**	0.13	0.93
		N	** < 0.0001**	** < 0.0001**	** < 0.0001**	**0.002**
		WA * N	**0.04**	0.45	0.37	0.96
FA	0	0	135 (8)^a^	12.3 (0.3)^a^	117 (8)^bc^	78 (6)^a^
		5	118 (6)^a^	15.3 (0.9)^a^	172 (13)^abc^	72 (4)^a^
		10	127 (7)^a^	13.4 (0.6)^a^	148 (21)^abc^	67 (4)^a^
		20	120 (10)^a^	15.7 (1.9)^a^	141 (11)^abc^	57 (9)^a^
	150	0	142 (8)^a^	12.2 (0.7)^a^	94 (11)^c^	55 (5)^a^
		5	129 (3)^a^	14.3 (0.7)^a^	213 (31)^a^	67 (8)^a^
		10	139 (8)^a^	14.0 (1.2)^a^	164 (27)^abc^	62 (11)^a^
		20	142 (12)^a^	13.7 (0.9)^a^	195 (16)^ab^	86 (6)^a^
		p values				
		WA	0.37	**0.01**	**0.0008**	0.75
		N	**0.03**	0.56	0.11	0.76
		WA * N	0.85	0.79	0.21	**0.01**

RCD, root collar diameter; SLA, specific leaf area.

Standard errors are shown in parentheses (n = 5). P values are from the result of two-way ANCOVA with initial RCD of seedlings as the covariate to detect the effect of wood ash and N fertilizer on height, RCD, leaf area, and SLA within each soil type. Significant p values are in bold. Different lower case letters within each soil indicate significant differences (p < 0.05, Tukey–Kramer comparison lines for least squares means) in each growth property across four wood ash levels and N fertilizer treatment.

Total leaf area and SLA were also affected by treatments and the response varied across soil types. Both wood ash and N fertilizer influenced total leaf area in LS soil, representing the highest value in 10 WA with N fertilizer. N fertilizer solely increased total leaf area by 1.7 times in FI soil while 5 WA increased total leaf area the most in FA soil. For SLA, there was no WA + N treatment effect in LS and FI soil but the interaction effect between WA and N fertilizer was observed in FA soil with the highest SLA in 20 WA + N.

**Figure 1:**
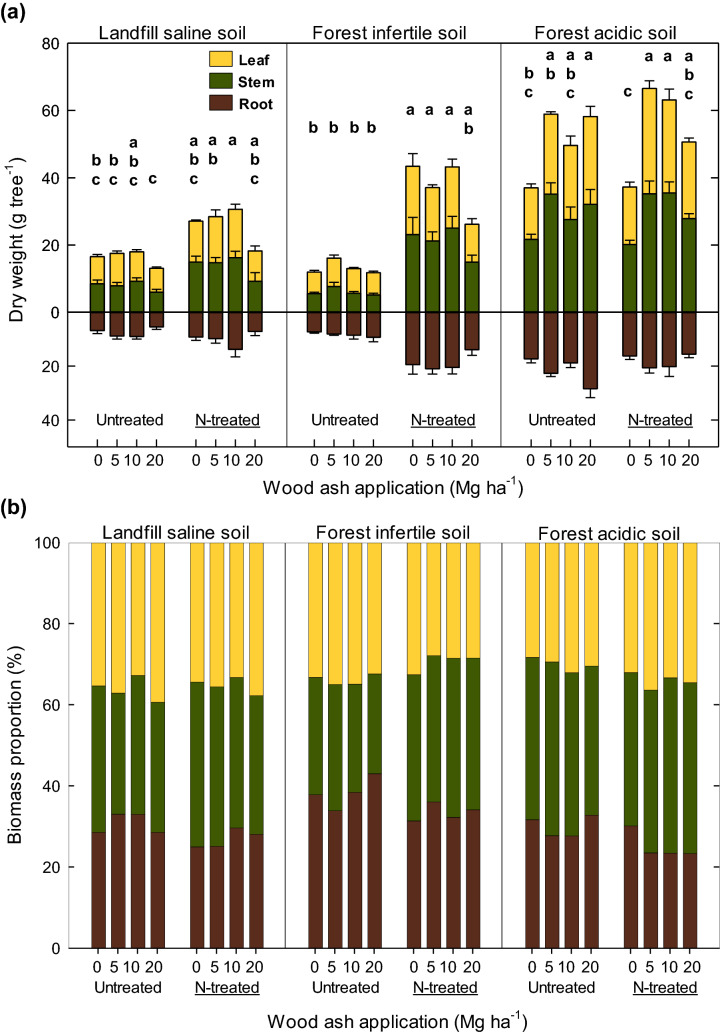
(**a**) Dry weight and (**b**) biomass proportion of aboveground (leaf and stem) and belowground (root) of *Zelkova serrata* seedling grown across three soil types (landfill saline soil, forest infertile soil, and forest acidic soil) applied with four levels of wood ash (0, 5, 10, and 20 Mg ha^−1^) and two levels of N fertilizer (0 and 150 kg ha^−1^). Vertical bars represent standard errors (n = 5). Different lower case letters within each soil indicate significant differences (p < 0.05, Tukey–Kramer comparison lines for least squares means) in each tissue across four wood ash levels and N fertilizer treatment.

Total plant biomass ranged from 19 to 87 g tree^−1^ across all soil types and it varied depending on treatments and soil types (Fig. 1a and Table S1). Average total biomass was the lowest in LS soil, followed by FI and FA soil. Both wood ash and N fertilizer had a significant effect on total and each organ biomass in LS soil. FI and FA soil responded differently to wood ash and N fertilizer treatment; the effect of N fertilizer was conspicuous in FI soil whilst wood ash treatment had more significant effect in FA soil. Particularly, N fertilizer improved more biomass production in FI soil than other soil types. In FA soil, WA application increased leaf, stem, and root biomass regardless of the applied amount, implying that 5 WA is enough to improve the biomass growth of the studied species by ameliorating soil acidity.

Biomass allocation pattern was also influenced by WA and N fertilizer treatments, and N fertilizer showed stronger influence than WA (Fig. 1b). N fertilizer decreased RS ratio by 17–21% across all soil types while WA application was only effective in FA soil. In addition, leaf proportion tended to increase with the amount of WA while root proportion seemed to be decreased by WA application except 20WA + N in FA soil.

### Foliage nutrient responses

Foliage nutrient concentrations of N, P, K, Ca, and Mg were more influenced by WA application than N fertilization, particularly in FA soil (Table 3). N fertilizer increased foliage N concentration in LS and FA soils while foliage P was decreased by N fertilizer in LS and FI soil. Contrarily, WA application increased foliage K in all soil types with the highest K concentration observed at 20 WA treatment except in FA soil without N fertilizer. Foliage Ca concentration seemed to increase as the amount of WA increased in LS and FI soil. Meanwhile, foliage Mg tended to be higher in the control than other treatments in all soil types.

**Table 3:** Foliage nutrient concentrations of seedlings applied with four levels of wood ash (WA; 0, 50, 10, and 20 Mg ha^−1^) and two levels of N fertilizer (N fert; 0 and 150 kg ha^−1^) across three different soil types (LS, landfill saline soil; FI, forest infertile soil; FA, forest acidic soil) at the end of this study (20 weeks after treatment).

Soil	N (kg ha^−1^)	Wood ash (Mg ha^−1^)	N (g kg^−1^)	P	K	Ca	Mg	Na
LS	0	0	16.84 (0.44)^bc^	2.20 (0.16)^a^	17.70 (0.74)^c^	6.79 (0.09)^b^	2.39 (0.16)^a^	0.165 (0.07)^a^
		5	13.78 (1.68)^c^	3.01 (0.47)^a^	17.31 (0.97)^c^	7.42 (0.31)^ab^	1.94 (0.21)^a^	0.204 (0.05)^a^
		10	19.51 (2.58)^abc^	3.01 (0.17)^a^	16.31 (0.85)^c^	12.11 (0.76)^a^	2.04 (0.05)^a^	0.195 (0.10)^a^
		20	19.97 (1.69)^bc^	2.74 (0.33)^a^	23.32 (2.85)^ab^	11.27 (0.26)^ab^	1.56 (0.15)^a^	0.050 (0.01)^a^
	150	0	26.14 (1.45)^a^	1.97 (0.22)^a^	19.15 (1.27)^bc^	7.22 (0.54)^ab^	2.50 (0.13)^a^	0.286 (0.18)^a^
		5	19.40 (0.36)^abc^	2.02 (0.22)^a^	16.55 (1.16)^c^	8.65 (0.97)^ab^	2.21 (0.15)^a^	0.413 (0.30)^a^
		10	23.50 (2.55)^ab^	2.06 (0.13)^a^	18.64 (0.38)^bc^	10.75 (1.78)^ab^	2.02 (0.27)^a^	0.052 (0.02)^a^
		20	25.31 (2.15)^ab^	2.12 (0.06)^a^	27.16 (2.24)^a^	11.19 (0.52)^ab^	1.86 (0.35)^a^	0.731 (0.61)^a^
		p value						
		WA	**0.03**	0.22	** < 0.0001**	**0.001**	0.05	0.54
		N	** < 0.0001**	**0.0003**	0.19	0.49	0.53	0.27
		WA * N	0.51	0.35	0.66	0.45	0.89	0.47
FI	0	0	14.53 (1.52)^a^	2.53 (0.29)^abc^	18.38 (0.95)^bc^	10.62 (0.36)^a^	0.70 (0.13)^a^	0.080 (0.01)^a^
		5	16.81 (3.71)^a^	2.61 (0.43)^abc^	20.57 (2.45)^ab^	10.95 (0.52)^a^	0.72 (0.18)^a^	0.066 (0.00)^a^
		10	13.46 (0.70)^a^	2.76 (0.22)^ab^	24.05 (1.46)^ab^	10.78 (1.27)^a^	0.60 (0.05)^a^	0.064 (0.02)^a^
		20	14.22 (0.96)^a^	3.31 (0.53)^a^	29.00 (2.30)^a^	9.76 (0.24)^a^	0.65 (0.08)^a^	0.056 (0.00)^a^
	150	0	13.27 (1.55)^a^	1.35 (0.02)^c^	10.68 (0.36)^c^	10.61 (0.64)^a^	1.09 (0.11)^a^	0.064 (0.02)^a^
		5	16.49 (0.78)^a^	1.58 (0.02)^bc^	19.20 (0.97)^abc^	10.73 (0.39)^a^	0.77 (0.05)^a^	0.060 (0.01)^a^
		10	15.90 (2.88)^a^	1.58 (0.12)^bc^	23.64 (3.50)^ab^	9.92 (0.26)^a^	0.86 (0.05)^a^	0.056 (0.01)^a^
		20	18.61 (2.10)^a^	2.07 (0.11)^abc^	27.89 (1.67)^a^	10.50 (0.36)^a^	1.09 (0.15)^a^	0.067 (0.01)^a^
		p value						
		WA	0.33	0.12	** < 0.0001**	0.81	0.25	0.69
		N	0.55	** < 0.0001**	0.09	0.63	**0.001**	0.53
		WA * N	0.70	0.98	0.18	0.79	0.31	0.59
FA	0	0	25.62 (1.22)^ab^	1.15 (0.07)^c^	11.03 (0.54)^c^	9.44 (0.57)^ab^	1.30 (0.10)^a^	0.076 (0.02)^a^
		5	21.18 (2.69)^ab^	1.44 (0.10)^abc^	20.66 (2.28)^a^	15.08 (2.41)^ab^	0.80 (0.08)^ab^	0.048 (0.00)^a^
		10	18.63 (1.27)^b^	1.84 (0.22)^ab^	18.60 (1.60)^ab^	14.31 (0.41)^ab^	0.85 (0.06)^ab^	0.053 (0.00)^a^
		20	19.27 (0.86)^b^	1.78 (0.17)^abc^	16.54 (1.67)^ab^	13.67 (0.35)^ab^	0.86 (0.04)^ab^	0.057 (0.01)^a^
	150	0	28.91 (2.32)^a^	1.22 (0.11)^bc^	12.32 (1.58)^bc^	8.00 (0.03)^b^	1.08 (0.23)^ab^	0.054 (0.00)^a^
		5	25.47 (1.63)^ab^	1.34 (0.09)^bc^	16.75 (0.96)^abc^	12.78 (0.55)^ab^	0.84 (0.10)^ab^	0.070 (0.01)^a^
		10	24.47 (1.85)^ab^	1.63 (0.04)^abc^	17.50 (0.54)^abc^	15.20 (2.34)^a^	0.72 (0.10)^b^	0.058 (0.00)^a^
		20	22.93 (1.23)^ab^	2.01 (0.12)^a^	19.86 (0.88)^ab^	12.42 (0.67)^ab^	0.86 (0.09)^ab^	0.048 (0.01)^a^
		p value						
		WA	**0.007**	**0.0002**	**0.0003**	**0.003**	**0.005**	0.23
		N	**0.002**	0.72	0.55	0.74	0.28	0.98
		WA * N	0.83	0.12	0.32	0.49	0.66	0.08

Standard errors are shown in parentheses (n = 3). P values are from the result of two-way ANOVA to detect the effect of wood ash and N fertilizer on each soil property within each soil type. Significant p values are in bold. Different lower case letters within each soil indicate significant differences (p < 0.05, Tukey–Kramer comparison lines for least squares means) for each nutrient concentration across four wood ash levels and N fertilizer treatment.

### Overall soil and plant responses to wood ash and N fertilizer treatment

**Figure 2:**
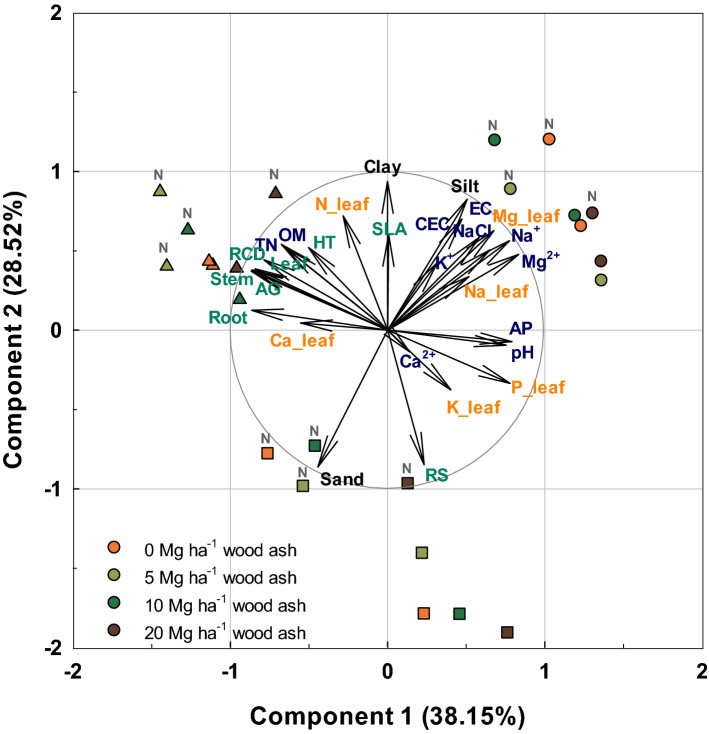
Principle component analysis (PCA) biplot based on the correlation matrix of soil characteristics and plant growth parameters across three soil types (landfill saline soil, forest infertile soil, and forest acidic soil) applied with four levels of wood ash (0, 5, 10, and 20 Mg ha^−1^) and two levels of N fertilizer (0 and 150 kg ha^−1^). Circle, square, and triangle represent landfill saline soil, forest infertile soil, and forest acidic soil. N above symbols denotes N fertilizer treated soil. OM, organic matter; TN, total nitrogen; AP, available phosphorus; CEC, cation exchange capacity, K, Ca^2+^ Mg^2+^, Na^+^, exchangeable cations; Stem, stem biomass; Leaf, leaf biomass; Root, root biomass; AG, aboveground (stem + leaf) biomass; RS, root to shoot ratio; HT, height; RCD, root collar diameter; N_leaf, P_leaf, K_leaf, Ca_leaf, Mg_leaf, Na_leaf, leaf nutrient.

The first, second, and third principal component accounted for 38.15%, 28.52%, and 12.19% of the total variation (totally 78.86%), respectively (Fig. 2 and Table S2). The first component seems to reflect overall effect of WA and N fertilization on soil chemical properties and plant growth because the first eigenvector shows approximately equal positive loading on soil pH, available P, exchangeable Mg^2+^, Na^+^, leaf P, Ca concentration and negative loading on soil organic matter, soil total nitrogen, stem, leaf, root biomass, and RCD growth (Table S3). The second eigenvector has high positive loadings on the variables soil silt and clay proportions but even higher negative loading on soil sand proportion, reflecting soil physical properties. The third eigenvector reflects the effect of wood ash because it has a very high positive loading on the variables WA application, exchangeable K^+^ and Ca^2+^ with subsequent effect on leaf nutrient concentration (high loadings on leaf K and Ca concentration).

All variables appeared to be divided into three groups according to initial soil types (Fig. 2). LS and FA soil positively correlated with the second principal component; however, LS soil positively correlated with the first principal component and FA soil negatively correlated with the first principal component. FI soil had low correlations with the first component but negatively related to the second component. Regardless of soil types, higher scores on the third component were related to high WA dose and it decreased with decreasing wood ash dose.

## Discussion

The results of this study showed that the magnitude of responses of soil chemical properties to treatments was different across soil types, supporting our first hypothesis. Specifically, we found that the pH of all soil types increased significantly after WA application; however, FA soil was more responsive to increasing amount of WA than LS and FI. The results can be attributed to the initial pH level of the soil used. Here the initial pH in FA soil was lower (i.e., 4.63) than that in LS (i.e., 6.88) and FI (i.e., 6.56), so WA application may have increased soil pH more in FA soil because of its higher number of hydrogen ions. A study reported that soil pH was more elevated for soils with low pH and low organic matter content^30^, although the FA soil used in this study had the highest organic matter content than other soils. In consistent with our results, Bang-Andreasen et al.^31^ observed the immediate increases in soil pH and EC after wood ash addition to the soil through their incubation experiment using the microcosm, and these observations are consistent with other studies showing that loose and fine-particulate wood ash is highly reactive in soils^32–34^. Also, wood ash has long been used in Scandinavian forests to correct nutritional deficiencies and acidity caused by whole-tree harvesting^35^. A meta-analysis by Augusto et al.^10^ reported no significant effect of wood ash on soil acidity status for short-term (in the first few years i.e., 1–5 years) on the mineral soil horizons, and the significant effect was detected in subsequent years (6–16 years). In contrast to that meta-analysis, we observed significant neutralization effect of wood ash on acidic soils even after one growing season (< 1 year) even at low ash doses (5 Mg ha^−1^ wood ash).

Although the pH of FA soil increased with increasing amount of WA, the generally acceptable pH increase should have been the addition of 5 Mg ha^−1^ to 10 Mg ha^−1^ wood ash because beyond these amounts, WA application resulted in a significantly higher pH (i.e. 8.2), which could be detrimental for plant growth, particularly species that are intolerant to high pH. However, at the end of the experiment, the biomass growth of FA-treated seedlings at the 20 Mg ha^−1^ WA was the highest among the WA alone treatments. The increase in root biomass was also found the highest at 20 Mg ha^−1^ WA. This pattern can be attributed to increase in CEC (14.7 cmolc kg^−1^) at 20 Mg ha^−1^ WA, which was also the highest among the other WA alone treatments. However, the foliar N, P, and K concentration of FA-treated plants were generally the highest at lower dosage treatments, i.e., 5 Mg ha^−1^ WA and/or 10 Mg ha^−1^ WA, and tended to decrease at 20 Mg ha^−1^ WA. This suggests that although a soil pH of 8.2 observed at 20 Mg ha^−1^ WA did not negatively affect the biomass growth of *Z. serrata*, the foliar N, P, and K concentrations may suffer from increasing the dosage of WA. Therefore, to make the use of WA as soil amendment environmentally-safe, 5 Mg ha^−1^ WA and/or 10 Mg ha^−1^ WA dosages may be safer because the growth of *Z. serrata* seedlings already showed a tendency to increase even at lower dosage compared with the control. Nevertheless, we could also imply that *Z. serrata* seedlings may be tolerant to high soil pH at WA alone treatment and the addition of N fertilizer may not be effective for seedling growth in acidic soil condition.

In the case of LS and FI soils, the highest dose of wood ash (20 Mg ha^−1^ WA) resulted in decreased seedling total and tissue biomass and this tendency was more significant in WA + N treatments. Our results also can be found in other studies and it may be attributed to excessive alkalization in soils of high wood ash dose resulting in decreased biomass production. Higher dose rates would likely have resulted in excessive alkalization and heavy metal loading of the soil^36^.

N application had an essential effect on most of the growth parameters for LS and FI soils. Here foliar N concentration was higher in N fertilized seedlings in LS soil but not in FI soil. Looking at the initial values, the total N of LS soil was significantly higher than that in FI soil, possibly indicating N limitation in FI soil even from the start of the experiment. Thus, the N fertilizer at N-deficient FI soil may have been fully used up for growth while that of LS soil can be considered as already sufficient for growth, thereby increasing the biomass growth of both LS- and FA-treated seedlings at the end of the N fertilization experiment. Results suggest that N fertilization is more effective than WA or WA + N application for fertilizing *Z. serrata* seedlings in LS and FI soil conditions. Consistent with our result, a positive plant growth was also observed when a nutrient-deficient municipal waste landfills were fertilized with N fertilizers^37^. Moreover, the favorable response of N fertilizer to most of the growth parameters for LS and FI soils can also be attributed to initial pH of these soils (6.56 – 6.88), which may have favored the availability of the other nutrients essential for plant growth, particularly during the first few weeks of the growing season or acclimatization phase.

Generally, wood ash did not solely enhance overall *Z. serrata* seedling growth but the mixture with N fertilizer was effective, particularly in infertile soil, although wood ash evidenced its capability of soil amendment in saline, infertile, and acidic soils in this study. This result partially supported our second hypothesis that adding N fertilizer would result in better plant growth performance by compensating the N deficiency in wood ash across all soil types. There have been a few available studies in which the effect of WA + N on tree growth across different soil types have been investigated. In a study of Jacobson^38^, there was also no significant difference in the stem growth between WA + N and N-alone treatments. Further, WA-alone treatment did not significantly affect tree growth in mineral soils^23,24^ but a contrasting result was observed when applied in other soil types^39^.

Overall, result implies that the growth of *Z. serrata* seedlings and foliar nutrients in FA soil can be improved by WA application even without adding N fertilizer and that of LS and FI by using only N fertilizer without WA. The effectiveness of WA in counteracting soil acidity, which in turn improved biomass growth of *Z. serrata* in FA soil, is a promising result relevant to restoring barren forest acidic soils or forest establishment in highly acidic lands in a sustainable way. As the demand for biomass-derived energy increases, the present study presented a reasonable approach on how to sustainably return the nutrients contained in ash back to the forest soil.

Results revealed also that utilization or amendment to increase the plant growth of *Z. serrata* seedlings varied by soil types, such that 5 Mg ha^−1^ WA and/or 10 Mg ha^−1^ WA for FA and only N fertilizer for LS and FI soil. This information is of high significance for tree growers and forest managers to efficiently select a site and soil amendment approaches for *Z. serrata.* Results of the study also revealed the effectiveness of WA (5 or 10 Mg ha^−1^) + N treatment in improving height growth of *Z. serrata* in infertile soil. This result can be beneficial for forest managers to effectively correct specific nutrient soil deficiencies in infertile lands to improve forest productivity (e.g., second rotation forests). We also found that *Z. serrata* seedlings can tolerate a high pH or basic soil condition. This provides us new insights on the potential of the species to restore or establish forest in alkali soils or areas characterized by large quantity of calcium carbonate (e.g., limestone forests).

Lastly, wood ash soil amendment seems to be feasible for restoring diminished nutrient pools in burnt forests caused by high intensity fires. Wood ash, which is the naturally produced residue remaining after forest fire, can catalyze the mineralization rates, through its influence on soil microorganism, and the increase in nutrient availability on a site via high nutrient load in ash. The application of wood ash in Canadian forests to mimic some of the effects of wildfire and replace nutrients removed has already been proposed^40^. Moreover, Woods and Balfour^41^ concluded that ash may significantly reduce the runoff and erosion in severely burnt forest, by providing the soil surface additional layer (1.5 cm thick) of protection from rainfall.

## Conclusion

In summary, wood ash without N is more responsive to soil chemical properties than N fertilizer and WA + N treatment in FA soil, whereas N fertilizer is more responsive to growth parameters than WA alone and WA + N treatment in LS and FI soil. Particularly, the application of over 5 Mg ha^−1^ of WA has shown to reduce soil acidity of FA soil, resulting in improved plant biomass production. In all soil types, the highest dose of WA (20 Mg ha^−1^) resulted in tree biomass decline, especially those under WA + N treatments. N fertilizer is more effective for improving plant growth, especially in LS and FI soil. Therefore, lower dosage of WA can be applied as a soil amender to counteract forest soil acidity and improve plant growth and foliar nutrient concentration, whereas N fertilizer can be added to correct nutrient soil deficiencies in landfill and infertile soils. This study should enhance our understanding of the effectiveness of WA in counteracting soil acidity, improving biomass growth, and correcting nutrient deficiency in forest soils in a sustainable way.

## Materials and methods

### Ethics statement

All necessary permits were obtained from the Forest Technology and Management Center, Korea Forest Service for the use of cultivated *Zelkova serrata* (Thunb.) Makino seedlings. We complied with ethical guidelines issued by the Center for Research Ethics Information in Korea. This study did not involve endangered or protected species.

### Study area and experimental design

This study was conducted in an experimental facility of the Forest Technology and Management Center, Korea Forest Service at Pocheon-si, Republic of Korea (37°45′N, 127°10′E). The experimental facility is designed to provide ambient environmental conditions, but the precipitation is intercepted by transparent panels. Mean temperature was 18.6 °C and relative humidity was 71.5% during the study period.

We conducted a 4 × 2 × 3 factorial experiment using a completely randomized design with five replications. Four levels of wood ash (0, 5, 10, and 20 Mg ha^−1^) and two levels of N fertilizer (0 and 150 kg ha^−1^) were applied across three different soil types: landfill saline soil, forest infertile soil, and forest acidic soil. Pots were randomly placed in eight parallel lines in the greenhouse following a 1 m distance between the lines and a 0.8 m distance between the pots. A total of 120 seedlings was used in this study. Manual watering was done three times per week and supplied 500 mL of water per pot.

### Preparation of experimental materials

#### Three different types of soils and their properties

We prepared three different types of soils originated from landfill area, urban forest area, and *Pinus rigida* Mill. plantation area and named each soil type as landfill saline (LS) soil, forest infertile (FI) soil, and forest acidic (FA) soil according to properties of each soil type (Table 4). LS soil was collected using a backhoe to a depth of 50 cm from landfill area close to the ocean where poplar trees were growing and herbaceous plants such as a common reed and green foxtail were naturally regenerating. FI soil was collected from an urban forest in the National Institute of Forest Science, Seoul, Republic of Korea. We removed the top organic layer and dug the soil to a depth of 30 cm. FA soil was collected from 40 years old *P. rigida* plantation in Namyangju-si, Republic of Korea. The top organic layer was carefully removed and then the soil was collected from a depth of 25 cm. All collected soils were transported to the study area, sieved through 10 mm in mesh size to remove stone and roots, and used for the experiment.

**Table 4:** Soil properties of three different soil types (landfill saline soil, forest infertile soil, and forest acidic soil) before fertilizer treatment.

Soil properties	Landfill saline soil	Forest infertile soil	Forest acidic soil
Sand (%)	1.0 (0.1)^c^	83.8 (0.5)^a^	52.1 (1.0)^b^
Silt (%)	81.0 (0.5)^a^	11.1 (0.5)^c^	32.9 (0.9)^b^
Clay (%)	18.0 (0.5)^a^	5.1 (0.0)^c^	15.0 (0.2)^b^
pH	6.88 (0.17)^a^	6.56 (0.09)^a^	4.63 (0.00)^b^
Organic matter (%)	0.95 (0.04)^b^	0.29 (0.01)^c^	3.77 (0.04)^a^
Total N (g kg^−1^)	0.46 (0.03)^b^	0.12 (0.00)^c^	1.06 (0.02)^a^
Available P (mg kg^−1^)	46.2 (1.0)^a^	19.6 (0.3)^b^	0.8 (0.2)^c^
CEC (cmol_c_ kg^−1^)	20.1 (0.3)^a^	8.5 (0.2)^b^	18.2 (0.8)^a^
Exchangeable K^+^ (cmol_c_ kg^−1^)	1.66 (0.06)^a^	0.19 (0.03)^b^	0.17 (0.02)^b^
Exchangeable Ca^2+^ (cmol_c_ kg^−1^)	1.04 (0.03)^b^	1.38 (0.05)^a^	0.25 (0.01)^c^
Exchangeable Mg^2+^ (cmol_c_ kg^−1^)	3.97 (0.07)^a^	0.81 (0.02)^b^	0.25 (0.01)^c^
Exchangeable Na^+^ (cmol_c_ kg^−1^)	5.65 (0.04)^a^	0.12 (0.01)^b^	0.14 (0.01)^b^
EC (dS m^−1^)	3.97 (0.34)^a^	0.22 (0.03)^b^	0.25 (0.00)^b^
NaCl (%)	0.18 (0.01)^a^	0.01 (0.00)^a^	0.01 (0.00)^b^

Standard errors are shown in parentheses (n = 3). Mean values with the same letters are not significantly different within each soil property across three soil types (p < 0.05; Tukey’s HSD test).

Three soil types were different physically and chemically (Table 4). In terms of soil texture, LS soil is silt loam, FI is loamy sand, and FA soil is sandy loam according to a proportional composition of clay, silt, and sand. Soil pH was remarkably lower in FA soil than others. Organic matter and total N were the highest in FA soil followed by LS and FI soil. Available P, EC, Exchangeable K^+^, Exchangeable Mg^2+^, and CEC were the highest in LS soil. Exchangeable Ca^2+^ was the highest in FI soil followed by LS and FA soil.

#### Wood ash and N fertilizer

Wood ash (WA) used in this study was generated from the combustion of oak wood and bark as a heating source. Generalizations on the properties of WA are difficult because the properties of wood ash varied depending on the source, type of waste, and combustion process^16,33,42^. However, WA generally has large porous particles of carbon, mineralized organic compounds, and oxidized basic cations according to available data previously reported. Most element concentrations of WA used in this study were lower than the previously reported values except N, K, Cu, and As (Table 5). N fertilizer applied with WA in this study was in the form of ammonium nitrate (NH_4_NO_3_) with 46% of N content.

**Table 5:** Element concentration in wood ash used in this study and that from reported values given as mean, a 95% confidence interval (CI), and median.

	This study	Reported values			
		Mean	95% CI	Median	n
Total N (g kg^−1^)	5.3 (0.3)	3.1	1.4	3.0	14
P (g kg^−1^)	5.0 (0.2)	11.27	0.9	10.0	214
K (g kg^−1^)	46.7 (1.7)	43.9	3.5	39.0	236
Ca (g kg^−1^)	52.5 (1.0)	165.8	11.4	160.0	227
Mg (g kg^−1^)	11.7 (1.2)	17.5	1.5	17.0	222
Na (g kg^−1^)	1.4 (0.1)	10.0	1.6	8.3	173
Al (g kg^−1^)	7.4 (1.4)	25.6	3.2	19.0	168
Fe (g kg^−1^)	3.3 (0.8)	22.1	5.6	12.0	206
Mn (g kg^−1^)	2.3 (0.3)	8.9	0.9	7.7	225
Cr (mg kg^−1^)	12.5 (1.3)	78.9	7.1	66.3	243
Ni (mg kg^−1^)	30.6 (2.1)	43.32	5.0	35.0	243
Cu (mg kg^−1^)	122.6 (9.7)	112.8	7.9	97.0	313
Zn (mg kg^−1^)	197 (50)	2157.1	307.9	1120.0	313
As (mg kg^−1^)	17.1 (0.2)	18.5	3.9	8.9	239

Cd and Pb were not detected in wood ash used in this study.

Standard errors are shown in parentheses (n = 3).

Mean, a 95% confidence interval, and median of reported values were calculated using the data of wood and/or bark fuel type ash in Wood Ash Database (http://woodash.slu.se).

#### Experimental treatment

We planted 1-year old Japanese zelkova (*Zelkova serrata*) seedlings in 35-L containers of three different types of soils collected from landfill area, urban forest, and pine plantation in late April. For experimental treatment, each soil of LS, FI, and FA was mixed with wood ash at four levels 0, 5, 10, and 20 Mg ha^−1^, which means 0, 69, 138, and 276 g for each container for wood ash treatment, respectively. To prevent soils from washing out of the container through the bottom hole, we placed a plastic sheet with a 2 mm in mesh size at the bottom and then put 1 cm size gravels approximately 5 cm in depth for good drainage in the container beforehand. We put the same amount of soils mixed with wood ash to the container and planted similar quality of vigorous seedlings produced in the same study area. Before planting seedlings, roots were cut at 8 cm in length and stem was cut at 20 cm in height. In mid-June, we applied two levels of N fertilizer 0 and 150 kg ha^−1^, which corresponds to 0 and 3.5 g for each container, and then irrigated to allow soil absorption of N fertilizer within each container.

### Measurement of seedling growth and biomass allocation

Initial root collar diameter (RCD) was measured at 3 cm above soil to examine the influence of initial RCD on seedling growth after treatments. At the end of growing season, we measured seedling height and RCD after seedling harvest. Also, we collected ten foliage for each seedling and measured leaf area using leaf area meter (LI-3100) to calculate specific leaf area (SLA, cm^2^ g^−1^) before tissue analysis.

We harvested seedlings carefully from the container and cut them into leaf, stem, and roots. Roots were thoroughly and cautiously washed with tap water to remove all soil particles on the root surface and not to drop fragile fine roots. The collected plant samples were oven-dried at 65 °C for 72 h and weighed for biomass estimation.

### Soil and foliar nutrient analyses

Soil samples (approximately 500 g) for physical and chemical analyses were collected at the start (before the treatment) and end of the study (after seedling harvest), respectively. For foliar nutrient analyses of N, P, K, Mg, and Na, we collected ten leaves from each seedling before seedling harvest. Soil and foliar nutrient analyses were conducted according to the National Institute of Agricultural Science and Technology^43^ and Sparks et al.^44^.

Soil texture was determined by Hydrometer method at 30 °C, and soil organic matter (OM) content was determined by wet combustion using Tyurin method^45,46^. Soil pH and electrical conductivity (EC) were measured using a 1:5 (w/v) soil: distilled water suspension. NaCl was calculated by multiplying conversion factor to EC. Total nitrogen (TN) was measured in 1 g soil by using the micro-Kjeldahl method^47^, and available phosphorus (P_2_O_5_; AP) was measured with Lancaster method^48,49^. The cation exchange capacity (CEC) was determined in 1 N HN_4_OAc and CH_3_COOH extracts by using the Brown method^50^. Exchangeable cations K^+^, Ca^2+^, Mg^2+^, and Na^+^ in the 1 N NH_4_OAc extract were determined using an Atomic Absorption Spectrometer (AA280FS, USA).

For foliage nutrient analysis, the oven-dried foliage was ground in a Wiley mill to pass through a 1-mm filter screen and organic matter was digested by using a block digester (BD-46, Lachat Ins., USA) with a combination of H_2_SO_4_ and HClO_4_. N and P concentrations were measured by the Automated Ion Analyzer (QuikChem AE, Lachat Ins., USA). K, Mg, Ca, and Na concentrations were determined by the Atomic Absorption Spectrometer (AA280FS, USA).

### Statistical analyses

Soil physical and chemical properties used in this study were subjected to one-way analysis of variance (ANOVA). The effect of wood ash and N fertilizer on soil properties of each soil was detected by two-way analysis of variance (ANOVA). In addition, significant differences in plant growth parameters were tested across wood ash levels and N fertilization for each soil type with two-way analysis of covariance (ANCOVA), with initial root collar diameter when planting as the covariate. For the significant results (p < 0.05), the mean values of soil and plant parameters were compared among wood ash applications by Tukey’s honestly significant difference (HSD) test or Tukey–Kramer method when the data are unbalanced. Principal component analysis (PCA) was performed to examine the association of effect of wood ash and N fertilizer with soil properties and plant growth parameters. The data analysis for this paper was generated using SAS 9.4 software. Copyright © 2017 SAS Institute Inc. SAS and all other SAS Institute Inc. product or service names are registered trademarks or trademarks of SAS Institute Inc., Cary, NC, USA.

## Supplementary Information

Supplementary Information.
