# Supplementary material for: Effects of wood ash and N fertilization on soil chemical properties and growth of Zelkova serrata across soil types
Source: Sci Rep. 2021 Jul 14;11:14489. doi: 10.1038/s41598-021-93805-5 (PMC8280140; doi:10.1038/s41598-021-93805-5)
Supplement: Supplementary file 1 — Supplementary Information. [file 41598_2021_93805_MOESM1_ESM.docx]

**Supplementary Material**

**Table S1** Result of two-way ANCOVA for the effect of wood ash and N fertilizer on leaf, stem, and root biomass and proportion across three different soil types (LS, landfill saline soil; FI, forest infertile soil; FA, forest acidic soil).

| Soil |  | p value |  |  |  |  |  |  |  |  |
| --- | --- | --- | --- | --- | --- | --- | --- | --- | --- | --- |
|  |  | Leaf | Stem | Aboveground | Root | Total | RS ratio | Leaf % | Stem % | Root % |
| LS | WA | **0.02** | **0.03** | **0.02** | 0.06 | **0.03** | 0.17 | 0.16 | 0.11 | 0.19 |
|  | N | **<0.0001** | **<0.0001** | **<0.0001** | **0.02** | **<0.0001** | **0.003** | 0.50 | **0.004** | **0.003** |
|  | WA*N | 0.44 | 0.42 | 0.41 | 0.76 | 0.57 | 0.36 | 0.73 | 0.24 | 0.35 |
|  |  |  |  |  |  |  |  |  |  |  |
| FI | WA | **0.03** | 0.11 | 0.05 | 0.27 | 0.08 | 0.16 | 0.57 | 0.39 | 0.19 |
|  | N | **<0.0001** | **<0.0001** | **<0.0001** | **<0.0001** | **<0.0001** | **0.0008** | **0.0006** | **<0.0001** | **0.0009** |
|  | WA*N | 0.18 | 0.51 | 0.36 | 0.27 | 0.38 | **0.04** | 0.15 | **0.045** | **0.03** |
|  |  |  |  |  |  |  |  |  |  |  |
| FA | WA | **<0.0001** | **<0.0001** | **<0.0001** | **0.01** | **<0.0001** | **<0.0001** | 0.27 | 0.24 | **0.0002** |
|  | N | **0.03** | 0.57 | 0.15 | **0.03** | 0.77 | **<0.0001** | **0.001** | 0.46 | **<0.0001** |
|  | WA*N | 0.10 | 0.30 | 0.21 | **0.02** | 0.12 | **0.02** | 0.33 | 0.56 | **0.03** |

RS ratio denotes root to shoot ratio. Aboveground denotes sum of leaf and stem. Total denotes sum of leaf, stem, and root.

Leaf%, Stem%, Root% denotes proportion of leaf, stem, and root to total biomass, respectively.

Significant p values are in bold.

**Table S2** Eigenvalues from a principle component analysis of the measured soil property values and plant growth parameters applied with four levels of wood ash and two levels of N fertilizer across three different soil types.

| Eigenvalues of the Correlation Matrix | | | | |
| --- | --- | --- | --- | --- |
|  | Eigenvalue | Difference | Proportion | Cumulative |
| 1 | 10.76 | 2.68 | 0.36 | 0.36 |
| 2 | 8.08 | 3.81 | 0.27 | 0.63 |
| 3 | 4.27 | 2.10 | 0.14 | 0.77 |
| 4 | 2.17 | 0.79 | 0.07 | 0.84 |
| 5 | 1.38 | 0.64 | 0.05 | 0.89 |
| 6 | 0.74 | 0.14 | 0.02 | 0.91 |
| 7 | 0.59 | 0.11 | 0.02 | 0.93 |
| 8 | 0.49 | 0.09 | 0.02 | 0.95 |
| 9 | 0.39 | 0.13 | 0.01 | 0.96 |
| 10 | 0.26 | 0.02 | 0.01 | 0.97 |
| 11 | 0.24 | 0.03 | 0.01 | 0.98 |
| 12 | 0.22 | 0.08 | 0.01 | 0.99 |
| 13 | 0.13 | 0.04 | 0.00 | 0.99 |
| 14 | 0.10 | 0.04 | 0.00 | 0.99 |
| 15 | 0.05 | 0.01 | 0.00 | 1.00 |

**Table S3** First five eigenvectors from the PCA of the measured soil property and plant growth parameter values applied with four levels of wood ash and two levels of N fertilizer across three different soil types.

| Eigenvectors of component order | | | | | |
| --- | --- | --- | --- | --- | --- |
|  | Prin1 | Prin2 | Prin3 | Prin4 | Prin5 |
| WA | 0.06 | -0.01 | 0.45 | -0.03 | -0.16 |
| N_Fert | -0.07 | 0.11 | 0.03 | 0.54 | -0.23 |
| Sand | -0.13 | -0.31 | 0.06 | 0.12 | 0.02 |
| Silt | 0.15 | 0.29 | -0.06 | -0.11 | -0.03 |
| Clay | -0.01 | 0.33 | -0.02 | -0.21 | 0.03 |
| pH | 0.23 | -0.02 | 0.22 | 0.19 | 0.26 |
| OM | -0.20 | 0.17 | 0.11 | -0.32 | -0.19 |
| TN | -0.21 | 0.18 | -0.04 | -0.27 | -0.22 |
| AP | 0.24 | -0.02 | 0.22 | 0.12 | -0.17 |
| K^+^ | 0.10 | 0.15 | 0.38 | -0.14 | -0.05 |
| Ca^2+^ | 0.05 | -0.05 | 0.46 | 0.09 | 0.06 |
| Mg^2+^ | 0.25 | 0.18 | -0.08 | 0.07 | 0.14 |
| Na^+^ | 0.23 | 0.21 | -0.09 | -0.01 | 0.14 |
| CEC | 0.17 | 0.21 | 0.03 | 0.02 | 0.12 |
| EC | 0.14 | 0.25 | 0.20 | -0.11 | 0.02 |
| NaCl | 0.20 | 0.18 | 0.03 | -0.05 | 0.29 |
| Stem | -0.27 | 0.12 | 0.10 | 0.06 | 0.14 |
| Leaf | -0.26 | 0.13 | 0.11 | 0.04 | 0.15 |
| AG | -0.26 | 0.13 | 0.11 | 0.05 | 0.15 |
| Root | -0.26 | 0.04 | 0.10 | 0.08 | 0.23 |
| RS | 0.08 | -0.30 | 0.00 | -0.09 | 0.04 |
| SLA | 0.00 | 0.22 | 0.02 | 0.31 | 0.10 |
| HT | -0.16 | 0.19 | 0.02 | 0.34 | -0.06 |
| RCD | -0.24 | 0.15 | 0.11 | 0.04 | 0.23 |
| N_leaf | -0.09 | 0.25 | -0.02 | -0.03 | -0.44 |
| P_leaf | 0.24 | -0.12 | 0.11 | -0.22 | 0.10 |
| K_leaf | 0.13 | -0.13 | 0.29 | 0.08 | -0.25 |
| Ca_leaf | -0.16 | 0.01 | 0.30 | -0.17 | 0.12 |
| Mg_leaf | 0.20 | 0.23 | -0.15 | 0.10 | 0.06 |
| Na_leaf | 0.15 | 0.13 | -0.01 | 0.15 | -0.35 |
